# Immunohistochemical Markers in Breast Cancer: A Cross-Sectional Study on Triple-Negative Breast Cancer in a Rural Tertiary Care Hospital

**DOI:** 10.7759/cureus.19486

**Published:** 2021-11-11

**Authors:** Rakesh BA, Venkata Pavan Kumar Karanam, Ashishkumar B Mundada

**Affiliations:** 1 General Surgery, DM Wayanad Institute of Medical Sciences (DM WIMS), Wayanad, IND; 2 General Surgery, Employees' State Insurance Corporation Medical College and Hospital (ESIC MCH), Hyderabad, IND

**Keywords:** triple-negative breast cancer, her2/neu protein, immunohistochemistry, progesterone receptor, estrogen receptor, breast cancer

## Abstract

Introduction

The incidence of breast cancer in India is on the rise, and it is now the most common cancer affecting women in India. The main objective of our study was to estimate the prevalence of triple-negative breast cancer (TNBC) in our study population and compare the various clinicopathological characteristics of TNBC with those of non-TNBC in these patients.

Methods

A retrospective, cross-sectional study was conducted among 249 cases of female breast cancer who reported to a tertiary care hospital in Southern India from September 2017 to September 2021.

Results

The mean age at presentation was 52 years (range: 26-82 years). The prevalence of triple-negative breast cancer was 19.7%. Most of the subjects belonged to the age group of 40-60 years. The majority were with grade 2 and 3 diseases. Of the cases, 50.6% were estrogen receptor (ER) positive and 48.2% were progesterone receptor (PR) positive, and 40.1% were HER2/neu positive.

Conclusion

The prevalence of triple-negative breast cancer in our study population is 19.7%, which is in concordance with the literature. Large tumor size, high-grade tumors, and a higher rate of axillary lymph node metastasis are characteristic features of TNBC. TNBC are tumors with aggressive tumor biology and are associated with poor prognosis.

## Introduction

The epidemiology of breast cancer across different population-based cancer registries in India are showing increasing trends for incidence and mortality, and breast carcinoma is now the most common cancer affecting women in India [1,2]. In the Indian population, the age-adjusted incidence rate is as high as 25.8 per 100,000 women, and the mortality is 12.7 per 100,000 women [2]. Breast cancer is a complex and heterogeneous disease that has been classified using numerous clinical and pathological features, including estrogen, progesterone, and HER2/neu receptor (epidermal growth factor receptor) expression. These features help in not only predicting the outcome but also determining the treatment strategy [3]. The College of American Pathologists and the American Society of Clinical Oncology have recommended the evaluation of estrogen receptor (ER), progesterone receptor (PR), and HER2/neu receptor for all newly diagnosed cases of invasive carcinoma [4].

Triple-negative breast cancer (TNBC) is defined as a breast cancer negative for all three receptors (ER, PR, and HER2/neu receptor). It has been shown to be an aggressive subtype with high rates of recurrence and poorer survival [5]. Currently, no targeted therapeutic agents are available specifically for TNBC subtypes. Studies that analyzed the response of tumors with neoadjuvant chemotherapy showed a good response in TNBC as compared with non-TNBC [6,7]. In this context, it is essential to be familiar with the features of TNBC to develop the best therapeutic approach.

The main objective of our study was to estimate the prevalence of TNBC in our study population and compare the various clinicopathological characteristics of TNBC with those of non-TNBC in these patients.

## Materials and methods

A retrospective, cross-sectional study was conducted among 249 cases of female breast cancer who reported to DM WIMS Medical College and Hospital catering to the rural population of a hilly area in Southern India from September 2016 to September 2021.

Patient characteristics

A detailed retrospective analysis was done using a proforma. Patient characteristics, such as age, menopausal status, and other clinical variables, were collected from the electronic medical records of the patients. Pathological characteristics, including breast cancer type, tumor size, tumor grade (modified Bloom-Richardson grade), axillary lymph node status (number of positive nodes), estrogen receptor status, progesterone receptor status, and HER2/neu status, were collected from the register available in the Department of Clinical Pathology Laboratory and also electronic medical records of the patients.

IHC methodology

Antigen retrieval was done using the BioGenex EZ-Retriever System (BioGenex Laboratories, CA, USA). ER status was assessed using monoclonal mouse IgG (clone 1D5), and PR status was assessed using monoclonal IgG1 (clone1A6). Receptor expression was considered positive when at least 1% of tumor nuclei stained positive for ER or PR. HER2/neu status was assessed using monoclonal IgG1 (clone CB11). A HER2/neu score of 3+ was considered positive by the immunohistochemistry method. Triple-negative breast cancer was defined as cancers that are ER negative, PR negative, and HER2/neu negative. Non-TNBC was defined as those that are positive for any of these markers. 

Statistical analysis

The data were entered and analyzed using IBM SPSS Statistics for Windows, version 27.0 (IBM Corp., Armonk, NY, USA). The frequencies and percentages of all variables were computed. A Chi-square (χ^2^) test was used to compare the association of the expression of ER, PR, and HER2/neu and the macroscopic and microscopic characteristics of the tumors. The results were considered statistically significant if the p-value was <0.05.

## Results

A total of 249 subjects were studied in the present study with a mean age of 52 years (range: 26-82 years). Most of the subjects belonged to the age group of 40-60 years (62.6%). Invasive ductal carcinoma (IDC) was found in 83.1% of the patients. The majority were with grade 2 and 3 diseases. The general clinicopathological characteristics of the study subjects are presented in Table 1.

**Table 1:** General clinicopathological characteristics of the study population N: number, IDC: invasive ductal carcinoma

Variable	N	%
Age group		
21–30	1	0.4
31–40	38	15.3
41–50	86	34.5
51–60	70	28.1
61–70	37	14.9
71–80	15	6
>80	2	0.8
Side		
Left	115	46.1
Right	126	50.7
Bilateral	8	3.2
Type		
IDC	207	83.1
Others	42	16.9
Grade		
1	26	10.4
2	135	54.3
3	88	35.3
Size		
T1	44	17.7
T2	164	65.9
T3	41	16.4
Lymph node status		
0	108	43.4
1–3	52	20.8
>3	89	35.8

**Figure 1:**
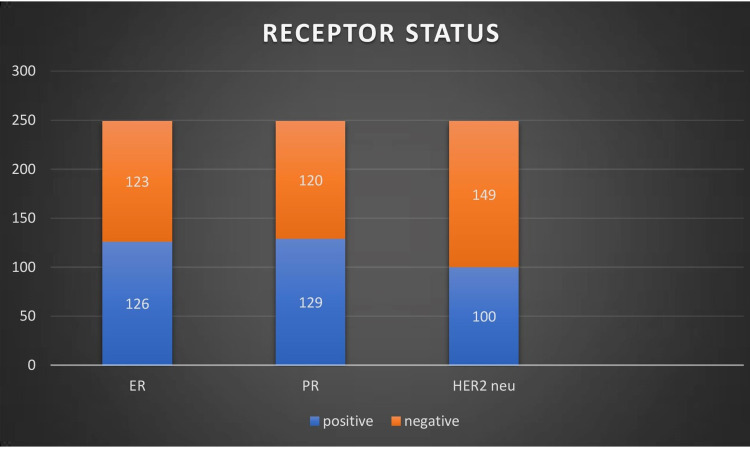
Receptor status of the patients studied ER: estrogen receptor, PR: progesterone receptor, HER2/neu: epidermal growth factor

Of the patients studied, 50.6% were ER positive, 48.2% were PR positive, and 40.1 % were HER2/neu positive (Figure 1).

**Figure 2:**
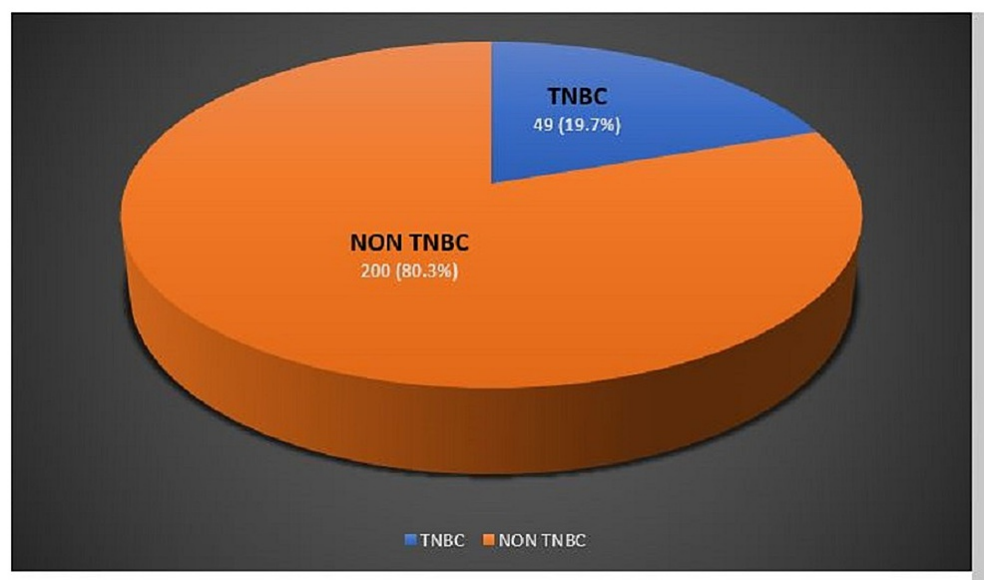
The prevalence of TNBC and non-TNBC among the study population TNBC: triple-negative breast cancer

Triple-negative breast cancer was noted in 19.7% (49 of 249) of the patients in the study (Figure 2).

Of the 49 patients with triple-negative breast cancer, 26 were younger than 50 years. A comparison of clinicopathological characteristics between patients with TNBC and non-TNBC is presented in Table 2.

**Table 2:** Comparison of clinicopathological characteristics of patients with TNBC and non-TNBC TNBC: triple-negative breast cancer, IDC: invasive ductal carcinoma p-value < 0.05 (statistically significant)

Variable	N-TNBC	TN	p-value
Age group			0.607
21 – 30	1	0	
31 – 40	30	8	
41 – 50	68	18	
51 – 60	57	13	
61 – 70	30	7	
71 – 80	12	3	
>80	2	0	
Side			
Left	97	12	0.745
Right	100	26	
Bilateral	3	5	
Type of Carcinoma			
IDC	165	42	0.538
Others	35	7	
Grade			
1	23	3	0.021
2	117	18	
3	60	28	
Size			
T1	40	4	0.042
T2	127	37	
T3	33	8	
Lymph Node status			
0	94	14	0.994
1 – 3	33	19	
>3	73	16	

## Discussion

With the increasing availability of facilities for IHC testing and also the affordable costs, the evaluation of estrogen receptor, progesterone receptor, and HER2/neu receptor status has become a standard routine in the management of breast carcinoma. The molecular heterogeneity of the disease signifies the prognosis and response to therapy. Hence, in the current study, we intended to estimate the prevalence of TNBC in our study population and compare the various clinicopathological characteristics of TNBC with those of non-TNBC in these patients.

The prevalence of TNBC in our study population was found to be 19.7%. As per the available literature, a higher prevalence of TNBC is observed in the Indian population than in Western populations [8]. However, the prevalence rates reported by various Indian studies showed considerable variation among different regions of the country. The prevalence of TNBC in our study was 19.7%, which is comparable with the studies of Ambroise et al. (25.5%) [9], Patnayak et al. (22.7%) [10], and Krishnamurthy et al. (18.5%) [11] and lower as compared with the data reported by Saha et al. (30.4%) [12], Rao et al. (50%) [13], and Zubeda et al. (46%) [14]. Early age of cancer onset, lifestyle factors such as diet and obesity, reproductive factors such as multiparity, socioeconomic factors, and potential genetic susceptibility of Indians to TNBC are the factors that might have accounted for the higher prevalence of TNBC reported by studies conducted among Indian patients. However, the data from the current study shows a prevalence similar to the West.

In the present study, the mean age at diagnosis was 52 years, which was consistent with previously reported data from India and a decade lower than that reported in the West [9-11,15]. This considerable difference might likely be due to the genetic, racial, and socioeconomic differences between the two populations. There is no statistically significant difference between mean ages at diagnosis in patients with TNBC (51.6 years) as compared with non-TNBC (52 years) in this study group.

Data from the study showed that 50.6% are ER positive and 48.2% are PR positive, which is low as compared with Western literature and is in concordance with previous Indian studies. Of the patients, 40.1% showed a HER2-positive status, which was higher as compared with the data in the literature [12-15].

In our study, 57.1% of the patients in the TNBC group had grade III disease, and only 30% of the patients in the non-TNBC group were diagnosed to have grade III disease. The difference is statistically significant (p = 0.021) and is also similar to that reported in the literature. A higher rate of lymph node positivity was noted in the TNBC group (71.4%) as compared with the non-TNBC group (53%) in our study [9-11]. The difference in lymph node metastasis between patients with TNBC and non-TNBC is statistically insignificant (p = 0.994), although lymph node metastasis is more common in the TNBC group in our study. This observation is consistent with similar conflicting reports on lymph node involvement in TNBC in the available literature. Patients with TNBC had relatively large tumor sizes compared with patients with non-TNBC (p = 0.042). Tumor size of more than 2 cm was noted in 91.8% of the patients with TNBC versus 80% of the patients with non-TNBC. These results are comparable with that of Dent et al. [16].

Our study has some limitations. The major limitations of the study are its retrospective design and small sample size. Large-scale prospective trials are required to ascertain rates of lymph node metastasis among both groups and identify a positive marker that can facilitate targeted therapy. Another major limitation is the lack of data regarding recurrence patterns and disease-free survival.

## Conclusions

Our study elaborated data on the immunohistochemistry profile of patients with breast cancer in this region of the country. The prevalence of triple-negative breast cancer is 19.7%, which is in concordance with the literature. TNBC are more common in India when compared with the Western world. Even in India, the incidence also varies from region to region. Large tumor size, high-grade tumors, and a higher rate of axillary lymph node metastasis are characteristic features of TNBC. Nevertheless, TNBC are tumors with aggressive tumor biology and are associated with poor prognosis.

## References

[1] Sathishkumar K, Vinodh N, Badwe RA (2021). Trends in breast and cervical cancer in India under National Cancer Registry Programme: an age-period-cohort analysis. Cancer Epidemiol.

[2] Malvia S, Bagadi SA, Dubey US, Saxena S (2017). Epidemiology of breast cancer in Indian women. Asia Pac J Clin Oncol.

[3] Prat A, Baselga J (2008). The role of hormonal therapy in the management of hormonal-receptor-positive breast cancer with co-expression of HER2. Nat Clin Pract Oncol.

[4] Hammond ME, Hayes DF, Dowsett M (2010). American Society of Clinical Oncology/College Of American Pathologists guideline recommendations for immunohistochemical testing of estrogen and progesterone receptors in breast cancer. J Clin Oncol.

[5] Carey L, Winer E, Viale G, Cameron D, Gianni L (2010). Triple-negative breast cancer: disease entity or title of convenience?. Nat Rev Clin Oncol.

[6] Liedtke C, Mazouni C, Hess KR (2008). Response to neoadjuvant therapy and long-term survival in patients with triple-negative breast cancer. J Clin Oncol.

[7] Carey LA, Dees EC, Sawyer L (2007). The triple negative paradox: primary tumor chemosensitivity of breast cancer subtypes. Clin Cancer Res.

[8] Sandhu GS, Erqou S, Patterson H, Mathew A (2016). Prevalence of triple-negative breast cancer in India: systematic review and meta-analysis. J Glob Oncol.

[9] Ambroise M, Ghosh M, Mallikarjuna VS, Kurian A (2011). Immunohistochemical profile of breast cancer patients at a tertiary care hospital in South India. Asian Pac J Cancer Prev.

[10] Patnayak R, Jena A, Rukmangadha N, Chowhan AK, Sambasivaiah K, Phaneendra BV, Reddy MK (2015). Hormone receptor status (estrogen receptor, progesterone receptor), human epidermal growth factor-2 and p53 in South Indian breast cancer patients: a tertiary care center experience. Indian J Med Paediatr Oncol.

[11] Krishnamurthy S, Poornima R, Challa VR, Goud YG (2012). Triple negative breast cancer - our experience and review. Indian J Surg Oncol.

[12] Saha A, Chattopadhyay S, Azam M, Sur PK (2012). Clinical outcome and pattern of recurrence in patients with triple negative breast cancer as compared with non-triple negative breast cancer group. Clin Cancer Investig J.

[13] Rao C, Shetty J, Kishan Prasad HL (2013). Morphological profile and receptor status in breast carcinoma: an institutional study. J Cancer Res Ther.

[14] Zubeda S, Kaipa PR, Shaik NA (2013). Her-2/neu status: a neglected marker of prognostication and management of breast cancer patients in India. Asian Pac J Cancer Prev.

[15] Rhodes A, Jasani B, Balaton AJ, Barnes DM, Miller KD (2000). Frequency of oestrogen and progesterone receptor positivity by immunohistochemical analysis in 7016 breast carcinomas: correlation with patient age, assay sensitivity, threshold value, and mammographic screening. J Clin Pathol.

[16] Dent R, Trudeau M, Pritchard KI (2007). Triple-negative breast cancer: clinical features and patterns of recurrence. Clin Cancer Res.

